# Mechanism of interaction between virus and host is inferred from the changes of gene expression in macrophages infected with African swine fever virus CN/GS/2018 strain

**DOI:** 10.1186/s12985-021-01637-6

**Published:** 2021-08-19

**Authors:** Bo Yang, Chaochao Shen, Dajun Zhang, Ting Zhang, Xijuan Shi, Jinke Yang, Yu Hao, Dengshuai Zhao, Huimei Cui, Xingguo Yuan, Xuehui Chen, Keshan Zhang, Haixue Zheng, Xiangtao Liu

**Affiliations:** State Key Laboratory of Veterinary Etiological Biology, National Foot-and-Mouth Disease Reference Laboratory, Lanzhou Veterinary Research Institute, Chinese Academy of Agriculture Science, Lanzhou, 73004 China

**Keywords:** African swine fever virus, Apoptosis, Chemotaxis, Innate immunity, Porcine alveolar macrophages, RNA-seq

## Abstract

**Background:**

African swine fever virus (ASFV) is a highly lethal virus that can infect porcine alveolar macrophages (PAMs). Since ASFV, China has dealt with a heavy blow to the pig industry. However, the effect of infection of ASFV strains isolated from China on PAM transcription level is not yet clarified.

**Methods:**

In this study, RNA sequencing (RNA-seq) was used to detect the differential expression of genes in PAMs at different time points after ASFV-CN/GS/2018 infection. The fluorescent quantitative polymerase chain reaction (qPCR) method was used to confirm the altered expression of related genes in PAMs infected with ASFV.

**Results:**

A total of 1154 differentially expressed genes were identified after ASFV-CN/GS/2018 infection, of which 816 were upregulated, and 338 were downregulated. GO and KEGG analysis showed that these genes were dynamically enriched in various biological processes, including innate immune response, inflammatory response, chemokines, and apoptosis. Furthermore, qPCR verified that the DEAD box polypeptide 58 (DDX58), Interferon-induced helicase C domain-containing protein 1 (IFIH1), Toll-like receptor 3 (TLR3), and TLR7 of PAMs were upregulated after ASFV infection, while TLR4 and TLR6 had a significant downward trend during ASFV infection. The expression of some factors related to antiviral and inflammation was altered significantly after ASFV infection, among which interferon-induced protein with tetratricopeptide repeats 1 (IFIT1)*,* IFIT2*,* Interleukin-6 (IL-6) were upregulated, and Ewing’s tumor-associated antigen 1 homolog (ETAA1) and Prosaposin receptor GPR37 (GPR37) were downregulated. In addition, we discovered that ASFV infection is involved in the regulation of chemokine expression in PAMs, and the chemokines, such as C-X-C motif chemokine 8 (CXCL8) and CXCL10, were upregulated after infection. However, the expression of chemokine receptor C-X-C chemokine receptor type 2 (CXCR2) is downregulated. Also, that the transcriptional levels of pro-apoptotic and anti-apoptotic factors changed after infection.

**Conclusions:**

After ASFV-CN/GS/2018 infection, the expression of some antiviral and inflammatory factors in PAMs changed significantly. The ASFV infection may activates the RLR and TLR signaling pathways. In addition, ASFV infection is involved in regulating of chemokine expression in PAMs and host cell apoptosis.

**Supplementary Information:**

The online version contains supplementary material available at 10.1186/s12985-021-01637-6.

## Background

The globalization of pig industry has promoted the emergence of infectious diseases affecting pigs and the spread of their pathogens, which is challenging for the healthy development of this industry. African swine fever (ASF) is an acute, febrile, highly contagious, and fatal animal infectious disease caused by ASF virus (ASFV) [1, 2], a large double-stranded DNA virus. The genomic length of different isolates varies from 170 to 190 kbp, encoding 151–167 open reading frames and > 170 proteins [3]. ASFV is the only member of the *Afarviridae* family and the only known insect-borne DNA virus that affects mammals [4–6]. It infects breeds of domestic pigs, African and Eurasian wild boars, and blunt ticks [7, 8]. Since 2018, a highly virulent type II ASFV has spread to China. ASFV has dealt a heavy blow to China, the world's largest producer and consumer of pork [9]. Although the experimental vaccine was produced by a natural, cell culture attenuated, or genetically modified ASFV, no effective vaccine has yet been produced.

Severa ASFV proteins have a major role in evading host defense and regulating host immune response by inhibiting interferon (IFN) production, apoptosis, and autophagy [6, 10–13]. For example, A238L and DP71L proteins regulate the host cell protein expression system that inhibits the host cell, shut down the expression system and inhibit the activation of transcription factors such as NAFT. The multigene family 360 (MGF360), MGF505/530 and I329L inhibit the anti-viral effect mediated by type I IFN; p54, DP71L, A179L, and A224L regulate apoptosis in the early and later stages of infection; A179L inhibits autophagy. In addition, DP96R of ASFV China/2018/1 negatively regulates type I IFN expression and nuclear factor-kappa B (NF-κB) signal transduction by inhibiting TBK1 and IKKβ [14]. The strong immune escape ability of ASFV makes it a powerful “killer”; thus, it is crucial to study the mechanism underlying the interaction with the host. Although some progress has been made in this research area, due to the large genome and complex structure of the virus, the exact mechanism underlying the interaction with the host is yet to be elucidated. The transcriptional analysis of host cell response to viral infection could be used to study the potential cytokines directly or indirectly related to viral infection and deduce the immune escape mechanism of the virus. RNA sequencing (RNA-seq) of transcriptome is a newly developed approach that can explore the mechanism of cellular signal transduction [15]. RNA-seq technique reveals the dynamic changes of the pathogen genome and the systematic changes in the host gene expression profile during pathogen infection [16, 17]. Previous studies have reported changes in the gene expression of PAMs infected with ASFV Malawi LIL20/1 isolate or ASFV Georgia 2007 strain [18, 19]. Currently, 13 ASFV strains are isolated from China, one of the major endemic places for ASFV. However, there is no report on the transcriptome of PAMs infected with ASFV China isolates.

In this study, RNA-seq was used to annotate host responses to ASFV-CN/GS/2018 strains isolated from China post-infection in PAMs. We also studied the differential gene expression of PAMs infected with ASFV-CN/GS/2018. The present study aimed to understand the host response at the various stages of ASFV-CN/GS/2018 infection at the cellular level, to provide a basis for an in-depth understanding of the biological mechanism of ASFV-host interaction, and to explain how ASFV infection regulates the host cell environment. These findings would contribute to the development of vaccines and other control strategies.

## Materials and methods

### Cell culture and virus

Porcine alveolar macrophages (PAMs) were prepared from bronchoalveolar lavage as described previously, cultured in Roswell Park Memorial Institute (RPMI) medium containing 10% porcine serum [20], and grown at 37 °C in a 5% CO_2_ atmosphere saturated with water vapor. ASFV-CN/GS/2018 is a virulent strain with genotype II, with no deletion of genes that inhibit the host response. The virus is provided by Lanzhou Veterinary Research Institute.

To determine the proliferation of the ASFV-CN/GS/2018 strains in PAMs, monolayers were prepared in 6-well plates and infected at multiplicity of infection (MOI) of 0.01 or 1. After 1 h of adsorption at 37 °C under 5% CO_2_, the inoculum was removed, and the cells were rinsed two times with phosphate-buffered saline (PBS). Then, the monolayers were rinsed with macrophage medium and incubated at 37 °C under 5% CO_2_ for different durations.

### Virus titration (50% hemadsorption doses)

The anticoagulant whole blood of healthy pigs is washed with sterilized PBS (pH 7.2) containing 1% gentamicin and centrifugation at 350×*g* for 3 min each time; subsequently, the PAMs are seeded in 96-well plate and the pig red blood cells are seeded in 96-well plate in a 20 μL volume. The sample was diluted at 10^–1^, 10^–2^, 10^–3^, 10^–4^, 10^–5^, 10^–6^, and 10^–7^, and plated in eight wells in a 96-well plate containing PAMs and red blood cells. The adsorption of red blood cells was observed for 7 days. Calculate 50% hemadsorption doses (HAD_50_) were calculated according to the Reed-Muench method.

### RNA-seq library preparation and Illumina sequencing

For cDNA library preparation, total RNA from the cell lines was treated with RNase-free DNase I (TaKaRa) following the manufacturer’s instructions. RNA was quantified using a NanoDrop ND1000 spectrophotometer (Thermo-Fisher Scientific), and the quality was assessed using a model 2100 Bioanalyzer (Agilent). The RNA integrity number value of each sample was > 8. The cDNA libraries were prepared according to the standard Illumina protocol (NEBNext® Ultra™ II RNA Library Prep Kit for Illumina®) and then subjected to sequencing using an Illumina HiSeq™ 2000 sequencer. The libraries were quantified using a DNA-1000 Kit Bioanalyzer (Agilent).

### Transcriptome assembly and transcriptional profiling analysis

After filtering the readings with sequencing connectors and low-quality readings, Hisat2 2.2.1.0 (RNA-strandness rf–fr) was used to align the remaining readings against the pig genome (Sscrofa11.1 GCF_000003025.6) and ASFV genome (GenBank: MK333180.1). HTSeq-count 0.9.1 (-s reverse) was used to analyze the reading distribution of known genes.

In order to analyze the gene level of PAMs infected with ASFV, the Cufflinks 2.1.1 (library-type fr-firststrand) program was used to quantify the fragments per kilobase in each million mapped readings of the genetic model (FPKM) to identify the genes in each cell. The false discovery rate (FDR)-corrected *P*-value < 0.05 was considered for differentially expressed genes (DEGs).

### Gene ontology (GO) enrichment and Kyoto Encyclopedia of Genes and Genomes (KEGG) pathway analysis

GO functional classifications were defined using the Blast2GO software. The enriched gene functional categories were further classified based only the GO analysis, *P*-value < 0.05. Kyoto Encyclopedia of Genes and Genomes (KEGG) pathway database was accessed using the KOBAS software via hypergeometric test, with a corrected p-value < 0.05. Q-value was used as a statistical method for estimating FDR, which is a conventional measure in the analysis of genome-wide expression data, with a corrected *P*-value < 0.05.

### Cell viability assays

The cell viability was measured using the cell counting kit-8 (CCK8) assay according to the manufacturer’s instructions. Briefly, the cells were seeded in 96-well plates and was infected with ASFV at 12, 24, and 36 h, respectively. Subsequently, 10 μL CCK-8 reagent (Apexbio) was added into each well, and after incubation at 37 °C for 2 h, the absorbance measured at 450 nm on a multifunction microplate reader (BioTek). The percentage at each concentration relative to the control was presented as cell viability.

### Real-time qPCR

Total RNA was extracted from PAMs using TRIzol reagent and reverse transcribed with PrimeScript RT kit (TaKaRa). qPCR was performed using a Power Up SYBR Green Master Mix on ABI StepOnePlus system, and data were analyzed by StepOnePlus software. The relative mRNA level of target genes was normalized to the porcine *GAPDH* mRNA level. The relative expression of mRNA was calculated based on the comparative cycle threshold (2^−ΔΔCT^) method [21].The Gene ID and primer sequence information are provided in Table 1.

**Table 1:** The gene ID involved in this study and the primers and oligonucleotides used

Primers	Sequences (5′–3′)	Gene ID
Porcine IL33-F	CTTCATGAGCAGCCCTCCAA	100518643
Porcine IL33-R	TCCGCAGCTTTCTGTCACAT	
Porcine IFITM3-F	CTGGTCCCTGTTCAACACCC	100518544
Porcine IFITM3-R	TGCAAACGATGATGAACGCAA	
Porcine BMP8A-F	CAGTCAGCACAGAAGTCCCC	100515668
Porcine BMP8A-R	CATCGAGGGTGTGTGTTCCT	
Porcine CDKN2B-F	CAAAGTGAGCGAGGAGGACAA	397227
Porcine CDKN2B-R	CAGAAGTTGACGCACGGTCT	
Porcine HPSE-F	AACCATAGACGGCAACCTGG	100271932
Porcine HPSE-R	TCTCAGGTATGCGGGAGACA	
Porcine MARCO-F	AAGGCCCACCAGGAATCAAG	100516298
Porcine MARCO-R	AAGTCACCTTTATGCCCCCG	
Porcine TLR3-F	ATGGATTGCTCCCCTTCACC	100037937
Porcine TLR3-R	CAGGGTTTGCGTGTTTCCAG	
Porcine TLR4-F	GACAGCAATAGCTTCTCCAGC	399541
Porcine TLR4-R	AAAGGCTCCCAGGGCTAAAC	
Porcine TLR6-F	TCTCATGGCACAGCGAACTT	396621
Porcine TLR6-R	TCACATCATCCTCTTCAGCGA	
Porcine TLR7-F	GCTGTTCCCACTGTTTTGCC	100037296
Porcine TLR7-R	ACTTGCGGTTGACTGAGGTT	
Porcine DDX58-F	GGAGATGCTTTCAGGGAGCG	396723
Porcine DDX58-R	GCAGTCTGGCCTAgCACAATA	
Porcine IFIH1-F	AGCCACAGATCAGCCAAGTC	100101927
Porcine IFIH1-R	TCCCATGGTGCCTGAATCAC	
Porcine IFIT1-F	TCCGACACGCAGTCAAGTTT	100153038
Porcine IFIT1-R	TGTAGCAAAGCCCTGTCTGG	
Porcine IFIT2-F	GCACAGCAATCATGAGTGAGAC	100155467
Porcine IFIT2-R	GGCCTGTATGTTGCACATCG	
Porcine IFITM3-F	CTGGTCCCTGTTCAACACCC	100518544
Porcine IFITM3-R	TGCAAACGATGATGAACGCAA	
Porcine RSAD2-F	AAAGACGTGTCCTGCTTGGT	396752
Porcine RSAD2-R	CTTCCGCCCGTTTCTACAGT	
Porcine ETAA1-F	TCTCAACAGCCAAAATGGCG	100622990
Porcine ETAA1-R	CGACTCATTGCCTAGGACCC	
Porcine IL-6-F	ACAAAGCCACCACCCCTAAC	399500
Porcine IL-6-R	CGTGGACGGCATCAATCTCA	
Porcine TNF-α-F	CCAGACCAAGGTCAACCTCC	397086
Porcine TNF-α-R	TCCCAGGTAGATGGGTTCGT	
Porcine NF-κB-F	CCCATGTAGACAGCACCACCTATGAT	751879
Porcine NF-κB-R	ACAGAGGCTCAAAGTTCTCCACCA	
Porcine GPR37-F	TTCCACGGTGACCAGTGATG	100523220
Porcine GPR37-R	ACAGAAGCGAACGTGGACAT	
Porcine CCL4-F	ATGAAGCTCTGCGTGACTGT	396668
Porcine CCL4-R	AGTCACGAAGTTGCGAGGAA	
Porcine CCL5-F	ACACCACACCCTGCTGTTTT	396613
Porcine CCL5-R	TGTACTCCCGCACCCATTTC	
Porcine CXCL8-F	AGCCCGTGTCAACATGACTT	396880
Porcine CXCL8-R	TGGAAAGGTGTGGAATGCGT	
Porcine CXCL10-F	ATAAGGATGGGCCGGAGAGA	494019
Porcine CXCL10-R	GTGGGAGCAGCTAACTTGGT	
Porcine CXCR2-F	GTGGAAACAGCAACTGCTCA	100124654
Porcine CXCR2-R	AGGGCTTGGTAGTTGTCAGG	
Porcine ISG12(A)-F	AGATACTGGCGACAGGGAGT	100153902
Porcine ISG12(A)-R	AGGGCAGCCTTGAATGACAG	
Porcine TNFSF10-F	TTGTGGAGCTCTGCCTGATG	406191
Porcine TNFSF10-R	ACCTTTCAGTGCTGCCCTTT	
Porcine GADD45B-F	GCCGCGGGTTCAGATTATTG	100621090
Porcine GADD45B-R	ACCTTCAGATCGCAGCGAAA	
Porcine IFI6-F	TCTGCTCTCTTCAAGGTCCG	110261124
Porcine IFI6-R	TCCACCGCAGGTGTAGAGTA	
Porcine PIK3CB-F	CTGCAGCTGGACGGTCG	100622559
Porcine PIK3CB-R	CCACTCACAATTTCACTGCCC	
Porcine HRK-F	ACGCTCTTTCATGTCTGGGG	100155596
Porcine HRK-R	CGTACAAACTGGCCCTGAGT	
Porcine GAPDH-F1	ACATGGCCTCCAAGGAGTAAGA	396823
Porcine GAPDH-R1	GATCGAGTTGGGGCTGTGACT	

Samples were collected at a specified time after PAM was inoculated with ASFV. Real-time quantitative PCR using ASFV P72 gene as a target to detect the copy number of ASFV genomic DNA. Using QIAamp® DNA Mini Kits (Qiagen, Germany) to extract sample DNA and then qPCR was carried out on a Bio-Rad system.

- ASFV-P72-R: 5′-CTGCTCATGGTATCAATCTTATCGA-3′;
- ASFV-P72-F: 5′-GATACCACAAGATCAGCCGT-3′;
- Taqman: 5′-CCACGGGAGGAATACCAACCCAGTG-3′.

Amplification conditions used were a preheating at 95 °C for 30 s and 40 cycles of 95 °C for 5 s and 58 °C for 30 s. The quantity of ASFV genome was calculated using the standard curve and expressed as genome copies per milliliter.

### Statistical analysis

The significance of the results between the experiments was analyzed using GraphPad Prism 8 (San Diego, CA, USA). All data are presented as mean values ± standard errors (SEs) from three independent experiments. *P < 0.05 was considered statistically significant. **P < 0.01 and ***P < 0.001 was considered highly statistically significant.

## Results

### Characteristics of PAMs infected with ASFV

**Fig. 1:**
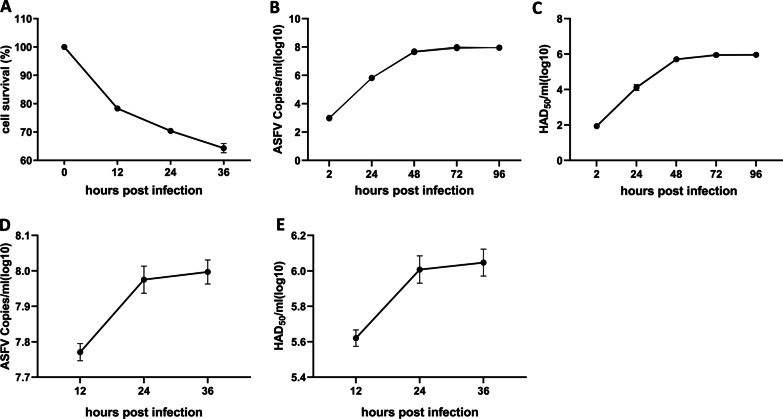
ASFV infection in PAMs. **A** The PAMs survival analysis after 12, 24, and 36 h of ASFV (MOI = 1) infection. **B**, **C** PAMs were infected by ASFV (MOI = 0.01), and the copy number (**B**) and titer (**C**) of the virus were determined at 2, 24, 48, 72, and 96 hpi. **D**, **E** PAMs were infected by ASFV (MOI = 1), and the copy number (**D**) and titer (**E**) of the virus were determined at 12, 24, and 36 hpi. Data are presented as mean ± SD of three independent experiments

PAMs are the natural host cell of ASFV, and are also the most widely used cell in ASFV related research. In Fig. 1A, with prolonged virus infection time, the activity of PAMs decreased gradually, which confirmed the high efficiency of ASFV infection.

In order to explore the proliferation kinetics of ASFV in PAMs, the viral load and viral titer in PAMs infected with ASFV (MOI = 0.01) for 0, 12, 24, 36, 48, 72, and 96 h were determined by qPCR and HAD_50_ methods. The results showed that the viral titer and DNA are increased gradually over a period post-infection and are maximal at 48 h after the infection (Fig. 1B, C).

In order to determine the exposure dose and infection time of RNA-seq samples, qPCR and HAD_50_ methods were used to detect the copy number and viral titer of ASFV in PAMs at 12, 24, and 36 h after ASFV (MOI = 1) infection. The results showed that the copy number and titer of ASFV (MOI = 1) reached a peak at 36 h post-infection (Fig. 1D, E).

### Evaluation of transcriptome sequencing data

We extracted total RNA and constructed sequencing libraries to perform deep sequencing. The Illumina-based RNA-seq was performed on HiSeq4000 platform. We analyzed and completed parametric transcriptome sequencing of 7 samples and obtained about 50.51 GB raw mRNA data. After screening, clean data of 47.64 GB were obtained, and the effective data of each sample were 6.20–7.00 GB, the Q30 base distribution was 93.48–94.45%, and the average GC content was 52.31% (Table 2). The sample reads were aligned to the reference genome, and the comparison rate was 93.60–98.07% (Table 3). We mapped the raw reads against the ASFV genome. The comparison rates of samples of 12, 24, and 36 h after PAM infection with ASFV were 1.16%, 3.71% and 2.03%, respectively (Table 4).

**Table 2:** Summary of RNA-Seq data

Sample	RawReads	RawBases	CleanReads	CleanBases	ValidBases (%)	Q30 (%)	GC (%)
MOCK 0 h	50.10M	7.52G	48.84M	7.00G	93.12	93.61	53.39
MOCK 12 h	48.40M	7.26G	47.35M	6.85G	94.29	93.82	51.76
MOCK 24 h	48.98M	7.35G	48.04M	6.90G	93.88	94.16	52.11
MOCK 36 h	44.18M	6.63G	43.06M	6.20G	93.52	93.48	52.44
ASFV 12 h	49.15M	7.37G	48.16M	6.92G	93.81	94.15	51.27
ASFV 24 h	49.81M	7.47G	48.82M	6.99G	93.54	94.45	52.95
ASFV 36 h	48.05M	7.21G	47.08M	6.78G	94.13	94.21	52.21

**Table 3:** Results of sequencing reads genome alignment

Sample	Total reads	Total mapped reads	Multiple mapped	Uniquely mapped
MOCK 0 h	48,840,654	47,432,538 (97.12%)	2,355,233 (4.82%)	45,077,305 (92.29%)
MOCK 12 h	47,349,948	46,434,174 (98.07%)	1,425,456 (3.01%)	45,008,718 (95.06%)
MOCK 24 h	48,044,790	47,087,739 (98.01%)	1,501,080 (3.12%)	45,586,659 (94.88%)
MOCK 36 h	43,057,476	42,163,761 (97.92%)	1,354,794 (3.15%)	40,808,967 (94.78%)
ASFV 12 h	48,162,550	46,579,302 (96.71%)	1,441,484 (2.99%)	45,137,818 (93.72%)
ASFV 24 h	48,823,380	45,698,836 (93.60%)	1,877,607 (3.85%)	43,821,229 (89.75%)
ASFV 36 h	47,081,798	44,956,457 (95.49%)	1,642,875 (3.49%)	43,313,582 (92.00%)

**Table 4:** Results of mapped the raw reads against the virus genome

Sample	Total reads	Total mapped reads	Multiple mapped	Uniquely mapped
ASFV 12 h	48,162,550	559,009 (1.16%)	184 (0.00%)	558,825 (1.16%)
ASFV 24 h	48,823,380	1,812,522 (3.71%)	1106 (0.00%)	1,811,416 (3.71%)
ASFV 36 h	47,081,798	954,599 (2.03%)	314 (0.00%)	954,285 (2.03%)

### Global changes in gene expression after ASFV infection

**Fig. 2:**
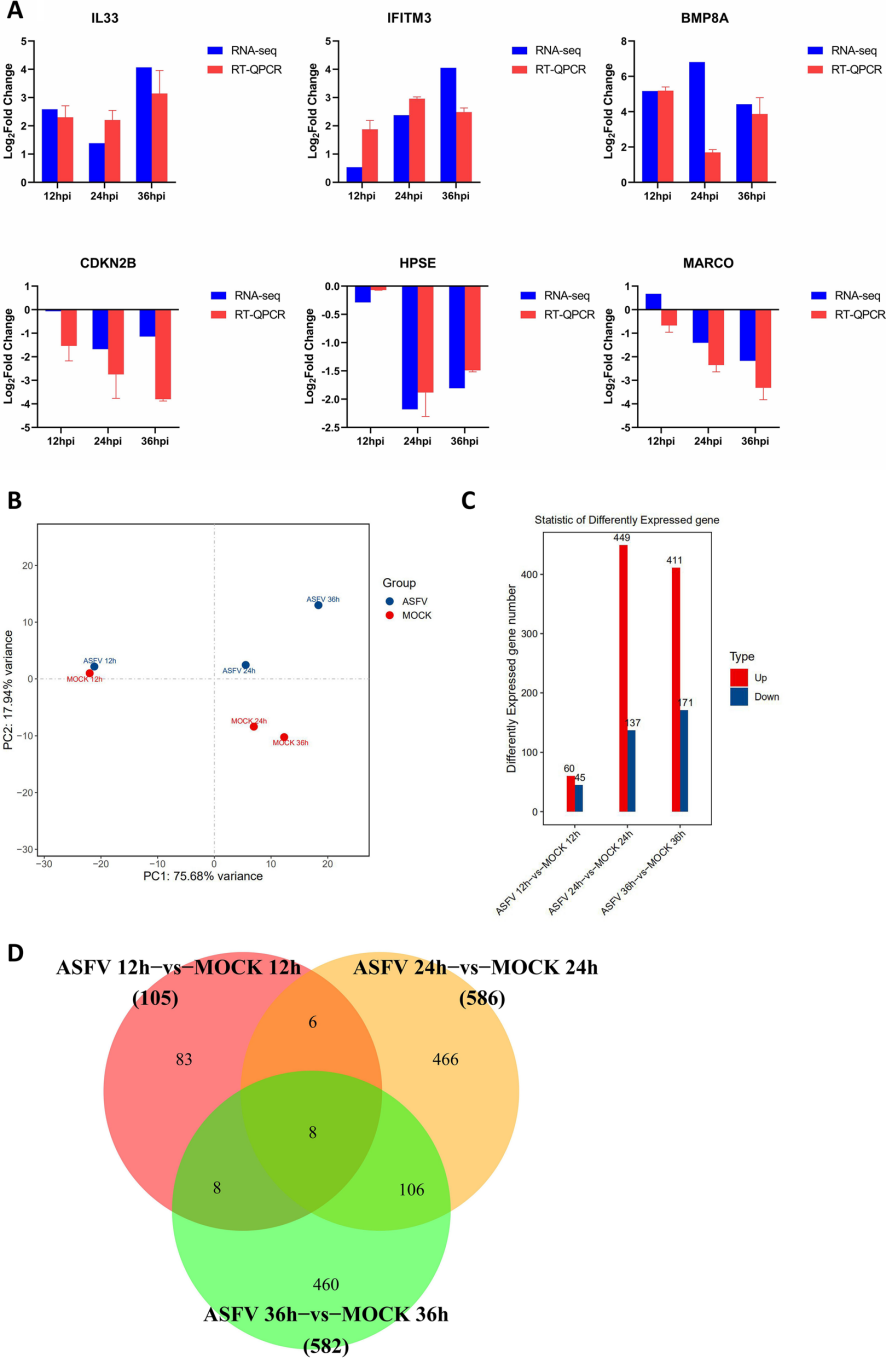
Changes in differential gene expression in PAM at different times after ASFV infection. **A** RT-qPCR vs. RNA-seq analyses of the expression for representative six genes (*IL33, IFITM3, BMP8A, CDKN2B, HPSE, and MARCO*). **B** The principal component analysis (PCA). **C** The upregulated/downregulated and the total number of DEGs (≥ 2 FC, *P* < 0.05) at 12, 24, and 36 h after PAMs infected by ASFV (MOI = 1). **D** Venn diagrams show an overlap of ASFV-induced DEGs across 12, 24, and 36 hpi. The fold-difference was measured by the 2^−ΔΔCt^ method. The RNA levels were normalized to corresponding GAPDH. Data are presented as mean ± SD of three independent experiments

Genes with ≥ twofold changes (FC) at 12, 24, and 36 h post-infection (hpi) were defined as DEGs (*P* < 0.05). In Fig. 2A, 3 up-regulated and 3 downregulated DEGs were randomly selected and verified by RT-qPCR; all showed good similarity with RNA-SEQ data, indicating that the RNA-SEQ data are accurate and valid that can be used for biological analysis. The principal component analysis (PCA) revealed the dissimilarities among different samples. Figure 2B shows a significant difference between mock-infected samples and ASFV-infected samples at 12, 24, and 36 hpi, and between mock-infected samples and ASFV-infected samples at 24 and 36 hpi (all *P* < 0.05 and |log2FC|> 1).

Next, the transcriptome profiles of the ASFV-infected and mock-infected groups at different time points were compared to determine the level of DEGs during ASFV infection. As shown in Fig. 2C, the number of DEGs (*P*-value < 0.05 and |log2FC|> 1) was 105, 586 and 582 at 12, 24 and 36 hpi, respectively, and most of the DEGs were upregulated or downregulated at 24 hpi. Among these, the 5 genes with the highest up-regulation or down-regulation at different time points (log2 FC) were screened for analysis (Table 5).

**Table 5:** The genes that were most up- or downregulated at each time point of ASFV infection

Gene category	12 hpi		24 hpi		36 hpi	
	Gene	log2Fold Change	Gene	log2Fold Change	Gene	log2Fold Change
Upregulated	BMP8A	5.17	BMP8A	6.81	HES4	9.37
	C2H19orf71	4.00	LOC110258836	6.62	IFIT1	6.70
	C1QTNF12	3.81	SCUBE2	5.84	PLAC8	6.49
	CHN1	3.70	FOXI1	5.54	RSAD2	6.45
	LOC100525318	3.46	LOC100521659	5.37	CXCL10	6.08
Downregulated	GPR37	− 3.70	AQP9	− 3.48	GPR37	− 4.10
	PAX5	− 3.00	HPSE	− 2.18	CUNH9orf116	− 3.74
	SFTPB	− 2.91	CXCR2	− 2.02	KCNE3	− 3.56
	GDPD1	− 2.75	CD209	− 2.00	SLC7A8	− 3.48
	SCNN1A	− 2.58	TLR6	− 1.81	LOC100622670	− 3.39

Among the upregulated genes, interferon-induced protein with tetratricopeptide repeats 1 (*IFIT1*) was significantly upregulated at all time points, and radical S-adenosyl methionine domain-containing protein 2 (*RSAD2*) expression was significantly upregulated at 24 and 36 hpi, indicating that antiviral and innate immune responses were activated. On the other hand, prosaposin receptor37 (*GPR37*) was significantly downregulated at 12 and 36 hpi, and aquaporin-9 (*AQP9*) was downregulated at 24 hpi (Table 3). The Venn diagram revealed the unique or shared genes at each time point (Fig. 2D). A total of 1154 DEGs were identified after ASFV infection; of these, 816 (71%) were upregulated, and 338 (29%) were downregulated. Furthermore, of the 816 genes, 60, 449, and 411 were upregulated at 12, 24, and 36 hpi, respectively. Among the 338 downregulated genes, 45, 137, and 171 were downregulated at 12, 24, and 36 hpi, respectively. The heat map of all DEGs at 12, 24 and 36 h after PAM infection is shown in Additional file 1. For all differential gene expression, see Additional file 2, Additional file 3 and Additional file 4.

### GO enrichment analysis

**Fig. 3:**
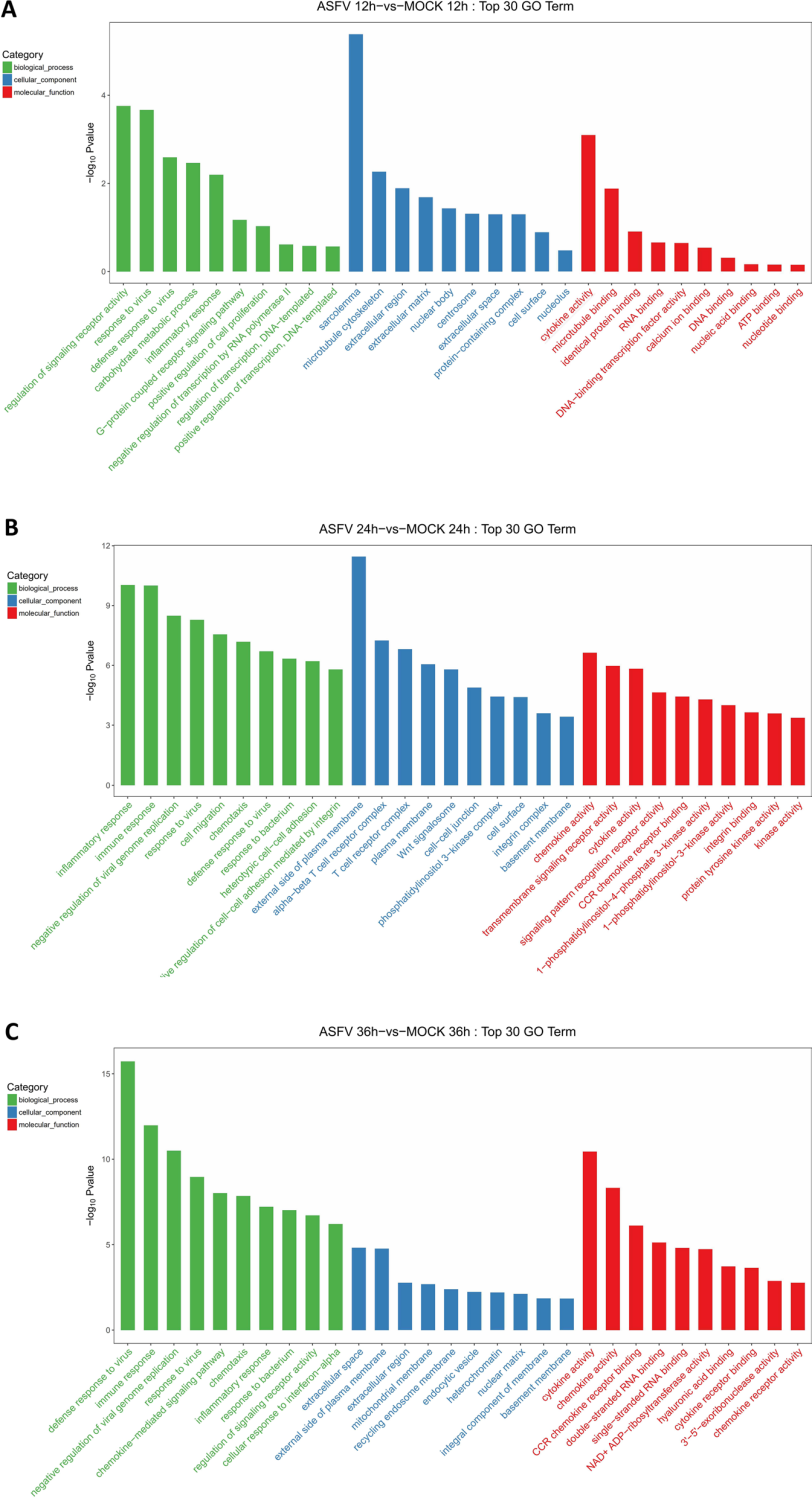
GO analysis of the genes with expression changes at 12, 24, and 36 hpi. **A** At 12 h after PAMs infected by ASFV (MOI = − 1), the top 30 GO-identified enriched DEGs. **B** At 24 h after PAMs infected by ASFV (MOI = − 1), the top 30 GO-identified enriched DEGs. **C** At 36 h after PAMs infected by ASFV (MOI = − 1), the top 30 GO-identified enriched DEGs

GO analyzed the DEGs in PAMs infected by ASFV at different time points to predict the biological function. The DEGs at 12 h after ASFV infection were mainly involved in the biological processes related to the regulation of signaling receptor activity, response to a virus, defense response to the virus, and the inflammatory response. Sarcolemma, microtubule cytoskeleton and extracellular region were enriched under the category of cellular components. Cytokine activity, microtubule-binding, and identical protein binding were enriched under the molecular function category (Fig. 3A). The DEGs at 24 hpi were mainly involved in the biological processes related to inflammatory response, immune response, negative regulation of viral genome replication, and response to the virus. The external side of the plasma membrane, alpha–beta T cell receptor complex, and T cell receptor complex were enriched under the cellular composition category, while chemokine transmembrane signaling receptor, and cytokine activities were enriched under the molecular function category (Fig. 3B). The DEGs at 36 hpi were mainly involved in the biological processes related to defense response to the virus, immune response, negative regulation of viral genome replication and response to a virus. The extracellular space, the external side of the plasma membrane, and the extracellular region were enriched under the cell composition category. Cytokine activity, chemokine activity, and CCR chemokine receptor binding were enriched under the molecular functional category (Fig. 3C).

### KEGG enrichment analysis

**Fig. 4:**
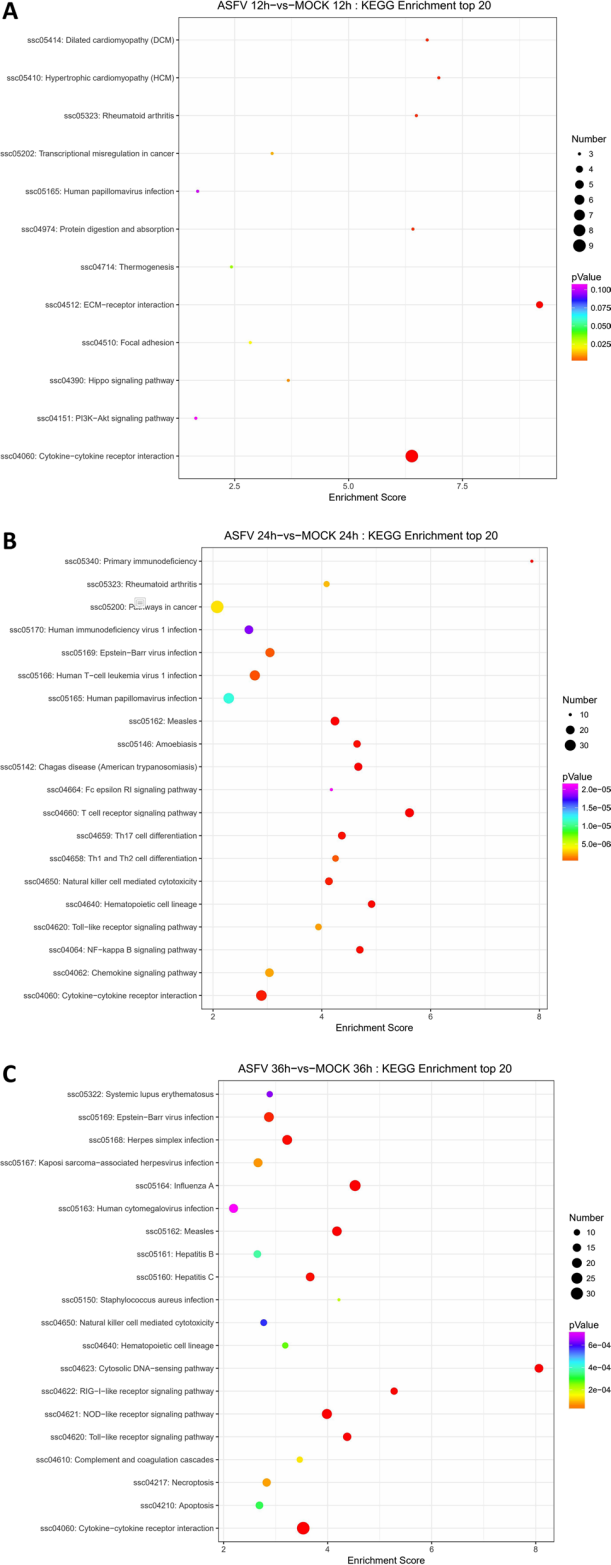
KEGG analysis of the genes with expression changes at 12, 24, and 36 hpi. **A** At 12 h after PAMs infection by ASFV (MOI = 1), KEGG enrichment analysis of top 20 DEGs. **B** At 24 h after PAMs infected by ASFV (MOI = 1), the top 20 KEGG enrichment analysis of DEGs.** C** At 36 h after PAM infected by ASFV (MOI = 1), the top 20 KEGG enrichment analysis of DEGs

KEGG enrichment analysis was carried out to further explain the individual function of the DEGs. We found that at 12 h after ASFV infection, DEGs were abundant in the extracellular matrix-receptor interaction and cytokine-cytokine receptor signaling pathways, with a key role in virus invasion, replication, and immune response. The DEGs were abundant at 24 hpi in cancer, T cell receptor, NF-kappa B signaling pathway, and cytokine–cytokine receptor interaction signaling pathways. DEGs at 36 hpi were abundant in RLR, NLR, and TLR signaling pathways. In addition, necroptosis and apoptosis-related genes were strongly enriched (Fig. 4C). These results suggest that ASFV infection may activates many types of pattern recognition receptors (PRRs).

### Transcriptional changes of PRRs in RLR and TLR pathways after ASFV infection

**Fig. 5:**
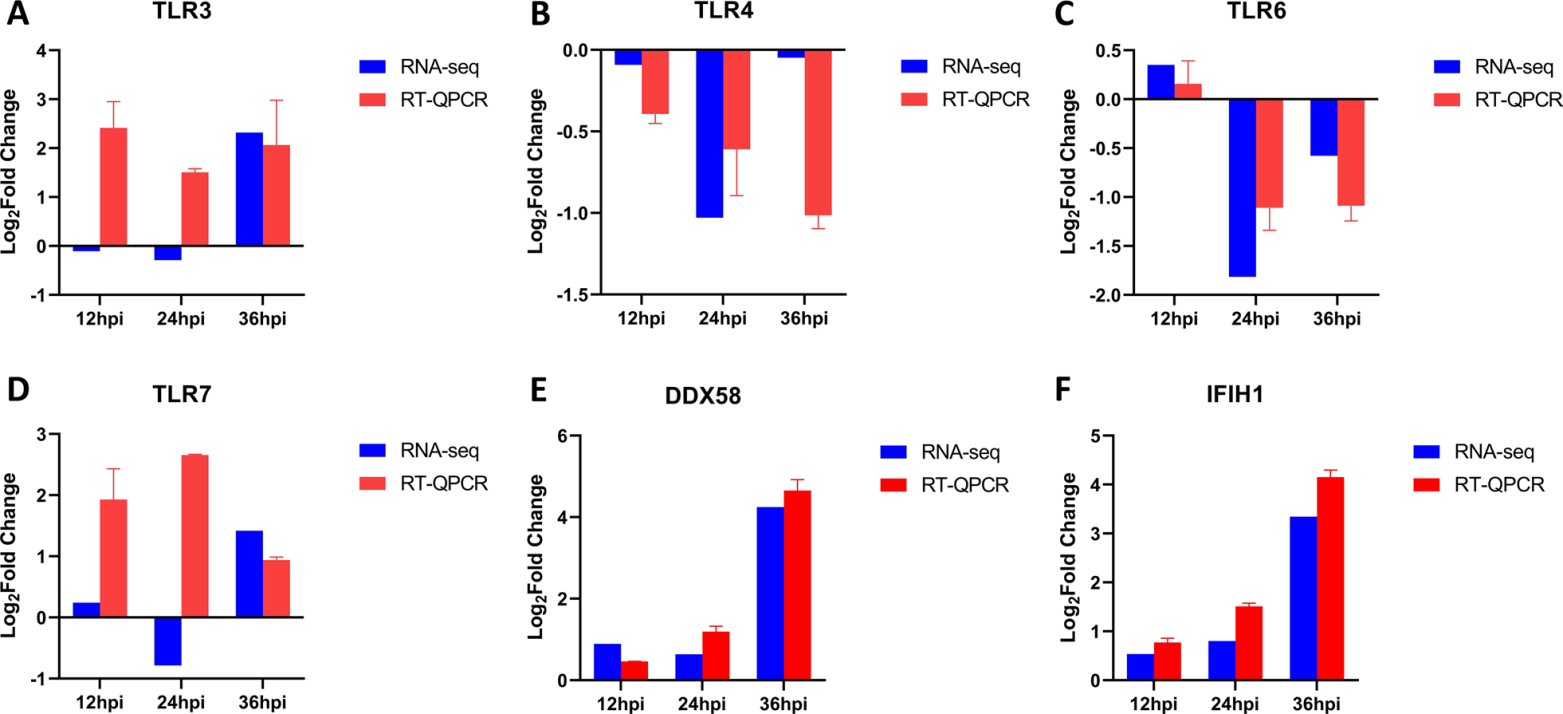
Transcription of DEGs related to RLR and TLR signaling pathways after ASFV infection. **A**–**F** At 12, 24, and 36 h after PAMs were infected with ASFV (MOI = 1), the transcriptional level of *TLR3* (**A**), *TLR4* (**B**), *TLR6* (**C**), *TLR7* (**D**), *DDX58* (**E**), and *IFIH1* (**F**) was detected by RT-qPCR. The fold-difference was measured by the 2^−ΔΔCt^ method. The RNA levels were normalized to corresponding GAPDH. Data are presented as mean ± SD of three independent experiments

Host PRR recognizes specific pathogen-associated molecular patterns (PAMPs), activating the innate immunity and exerting an anti-viral role [22]. In KEGG enrichment analysis, DEGs are significantly enriched in the RLR and TLR signaling pathways (Fig. 4B, C). Therefore, the transcriptional level of *TLR3, TLR4, TLR6, TLR7, DDX58, and IFIH1* was evaluated. We observed that the transcriptional level of *TLR3* (Fig. 5A)*, TLR7* (Fig. 5D)*, DDX58* (Fig. 5E)*,* and *IFIH1* (Fig. 5F) was upregulated at different time points and *TLR4* (Fig. 5B) and *TLR6* (Fig. 5C) were negative regulators of the RLR pathway with a significant downward trend during ASFV infection. These data suggested that ASFV infection may activates the RLR and TLR signaling pathway.

### Transcription changes of antiviral-, inflammatory-, and chemotactic-related factors in PAMs infected with ASFV

**Fig. 6:**
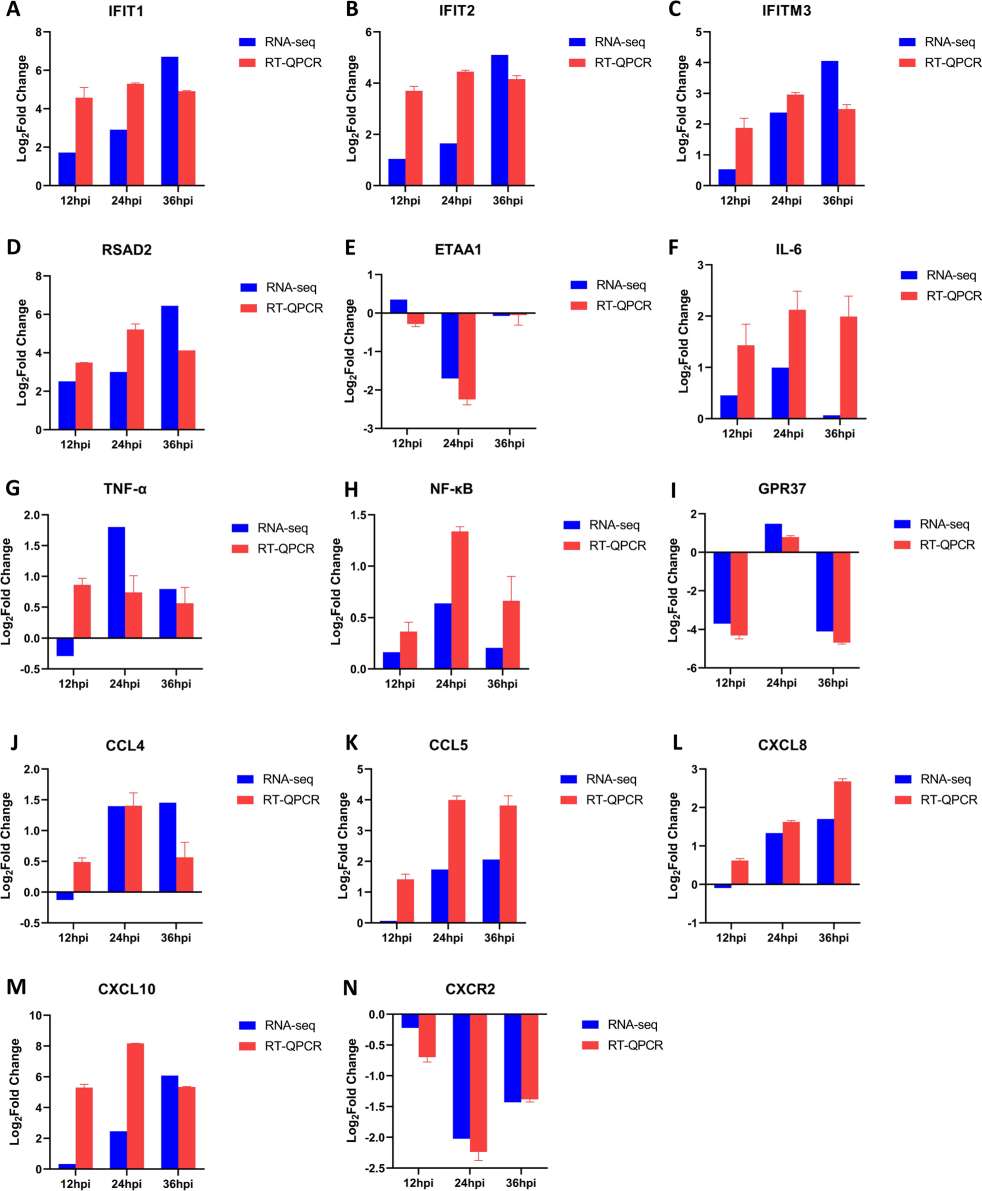
Effect of ASFV infection on the transcription of anti-viral factors, inflammatory, and chemokines. **A**–**N** At 12, 24, and 36 h after PAMs were infected by ASFV (MOI = 1), the transcriptional level of *IFIT1* (**A**), *IFIT2* (**B**), *IFITM3* (**C**), *RSAD2* (**D**), *ETAA1* (**E**), *IL-6* (**F**), *TNF-α* (**G**), *NF-κB* (**H**), *GPR37* (**I**), *CCL4* (**J**), *CCL5* (**K**), *CXCL8* (**L**), *CXCL10* (**M**) and *CXCR2* (**N**) were detected by qPCR. The fold-difference was measured by the 2^−ΔΔCt^ method. The RNA levels were normalized to corresponding GAPDH. Data are presented as mean ± SD of three independent experiments

In GO and KEGG enrichment analysis, DEGs were significantly enriched in anti-viral infection, innate immunity, and inflammation-related pathways (Figs. 3, 4). Therefore, the transcriptional levels of anti-viral and inflammation-related factors were evaluated further after ASFV infection. The transcriptional levels of anti-viral and inflammation-related factors were detected after ASFV infection. The results of qPCR and RNA-seq showed that ASFV infection upregulated the transcriptional levels of anti-viral related factor *IFIT1* (Fig. 6A)*, IFIT2* (Fig. 6B)*,* Interferon-induced transmembrane protein 3 (*IFITM3*) (Fig. 6C)*, RSAD2* (Fig. 6D), inflammation related factor *IL-6* (Fig. 6F), Tumor necrosis factor α (*TNF-α*) (Fig. 6G), Nuclear factor NF-kappa-B p50 subunit (*NF-*κ*B*) (Fig. 6H) to different degrees. The transcriptional levels of *ETAA1* (Fig. 6E), a factor related to immune cell function, and *GPR37* (Fig. 6I), a factor related to inflammation and macrophage polarization, were downregulated to varying degrees after ASFV infection.

PAMs are an immune cells with strong chemotaxis, can secrete a variety of chemokines, and attract and activates immune cells to enhance the immune and inflammatory response [23]. Therefore, combined with the data of RNA-seq, the transcriptional level of *CCL4* (C-C motif chemokine 4)*, CCL5, CXCL8,* and *CXCL10* in PAMs infected with ASFV was detected by qPCR at different time points. The transcriptional level of *CCL4* (Fig. 6J)*, CCL5* (Fig. 6K)*, CXCL8* (Fig. 6L), and *CXCL10* (Fig. 6M) was upregulated to varying degrees, while that of *CXCR2* (Fig. 6N) was significantly downregulated after ASFV infection. Overall, the transcription of chemokine in PAMs was upregulated by ASFV infection.

### Differential expression of apoptosis-related cytokines in ASFV-infected PAMs

**Fig. 7:**
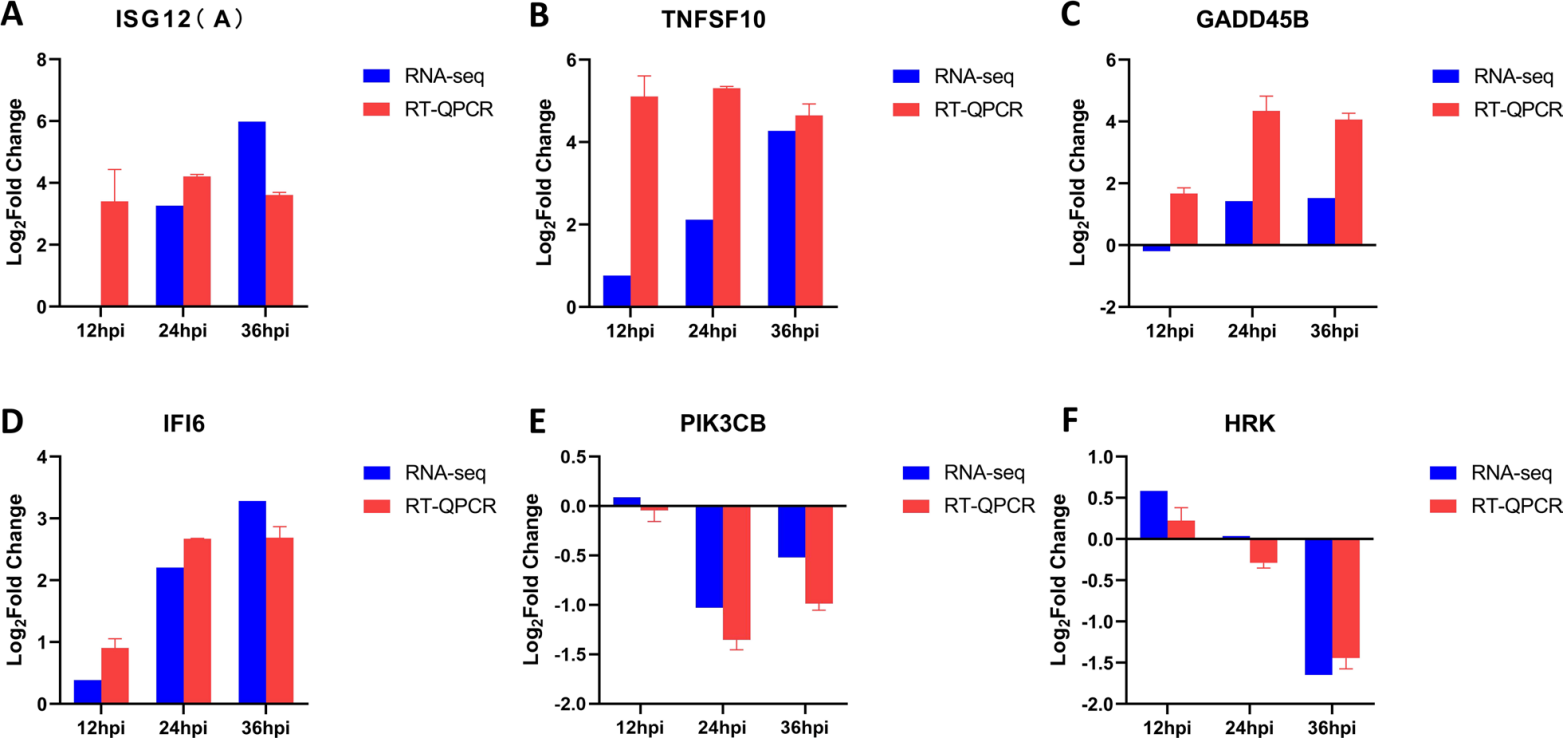
Differential expression of apoptosis-related cytokines in PAM infected by ASFV. **A**–**D** At 12, 24, and 36 h after PAM infection with ASFV (MOI = 1), the transcriptional level of *ISG12* (**A**), *TNFSF10* (**B**), *GADD45B* (**C**), *IFI6* (**D**)*, PIK3CB* (**E**) and *HRK* (**F**) was detected by qPCR. The fold-difference was measured by the 2^−ΔΔCt^ method. The RNA levels were normalized to corresponding GAPDH. Data are presented as mean ± SD of three independent experiments

After infecting the host, many viruses regulate host apoptosis, which in turn promotes the replication of the virus. In KEGG enrichment analysis (Fig. 4C), DEGs were enriched in the apoptotic pathway. Combined with the data of RNA-seq, the transcription level of cytokines related to apoptosis was verified, and we found that *ISG12 (A)* (Fig. 7A), Tumor necrosis factor ligand superfamily member 10 *(TNFSF10*) (Fig. 7B), *GADD45B* (Fig. 7C), which promotes apoptosis, and Interferon alpha-inducible protein 6 (*IFI6*) (Fig. 7D), which negatively regulates apoptosis, were upregulated after ASFV infection. In addition, phosphatidylinositol 4, bisphosphate 3-kinase catalytic subunit beta isoform (*PIK3CB*) (Fig. 7E) and activator of apoptosis hara-kiri (*HRK*) (Fig. 7F), which promote apoptosis [24, 25], were downregulated after ASFV infection. Thus, it could be speculated that ASFV infection may be involved in the regulation of host apoptosis in many ways.

## Discussion

ASFV is harmful pathogen to pigs. Since 2018, it has caused huge economic losses to the pig industry in China [9]. Macrophages are major target cells of ASFV, and they are also important immune cells of the host [26]. In addition, macrophages also trigger acquired immunity. Therefore, an in-depth insight into the transcriptional changes of ASFV-infected macrophages is crucial to understand the host–pathogen interaction. With the continuous development of new technology, RNA-seq is a major tool to elucidate the transcriptional spectrum [27]. Previous studies have applied RNA-seq technology to transcriptome studies in pigs infected with highly virulent (Georgia 2007 strains) or low virulent (OURT33) ASFV [28]. Some studies have revealed the altered of gene expression in PAMs infected with ASFV Georgia 2007 strain within 18 hpi [19]. One replication cycle of ASFV is about 16 h, and infectious offspring virus can be produced at 16 hpi. We selected three time points to collect samples: 12, 24 and 36 hpi. In the present study, we used PAMs as an in vitro model and analyzed the transcriptional changes of host cells infected with ASFV-CN/GS/2018 strain using RNA-seq technique. A total of 1154 DEGs were identified, of which 816 genes were upregulated, and 338 genes were downregulated (Fig. 2C). The KEGG enrichment analysis of DEGs found that TLR and RLR signaling pathways may be involved in response to ASFV infection (Fig. 4B, C). Subsequent qPCR verification found that the transcription of *TLR3* (Fig. 5A)*, TLR7* (Fig. 5D)*, DDX58* (Fig. 5E)*,* and *IFIH1* (Fig. 5F) was upregulated, which further suggested that TLR and RLR signal pathways may be activated after ASFV infection. *TLR3* mainly recognizes dsRNA; *TLR7* primarily identifies ssRNA and a few short dsRNA; *DDX58* identifies dsRNA and 5Powerppp ssRNA, while *IFIHI* identifies dsRNA with a length > 1 kbp. Reportedly, some DNA viruses, such as herpesvirus infection, can activate the RIG-I signaling pathway [29]. HSV-1 infection increases the content of RNA5SP141 in the cytoplasm and downregulates proteins that bind to RNA5SP141, which in turn binds RNA5SP141 to RIG-I and induces type I interferon [30]. However, additional studies are required to identify whether and how the sensor pathway of RNA is involved in the infection process of ASFV, a DNA virus.

Also, in this study, the transcriptional levels of downstream anti-viral and inflammatory factors were analyzed further, and the results of RNA-seq and qPCR showed that ASFV infection could upregulate the transcriptional level of *IFIT1* (Fig. 6A)*, IFIT2* (Fig. 6B)*, IFITM3* (Fig. 6C)*, RSAD2* (Fig. 6D), *IL-6* (Fig. 6F), *TNF-α* (Fig. 6G) and *NF-κB* (Fig. 6H). The activation of these factors indicates that the PAMs is in an anti-viral state, which is verified with the activation of the above immune-related pathways. However, the differential expression of anti-viral and inflammatory factors revealed that the immune and inflammatory activation of PAMs infected with ASFV was very limited. We speculated that after virus infection, immune and inflammation-related pathways are activated and then suppressed by a large number of immune escape proteins encoded by ASFV. In addition, the transcriptional levels of *ETAA1* (Fig. 6E) and *GPR37* (Fig. 6I) are downregulated during ASFV infection. Previous studies have shown that removing a gene called *ETAA1* from mice prevents the animal from producing an immune response to vaccines or infections [31]. Mice without *GPR37* showed delayed phagocytosis of macrophages and a delayed regression of inflammation. At the cellular level, macrophages without *GPR37* gene showed an imbalance of anti-inflammatory and pro-inflammatory cytokines [32]. Another important role of *GPR37* is to regulate the phenotype of macrophages. Macrophages expressing *GPR37* show more M2 than M1 [32].

Macrophages produce chemokines that induce pathology and protective immunity and play a key role in anti-viral response [33]. Additionally, some large DNA viruses, such as herpesvirus and poxvirus, can regulate chemokine activity by encoding homologs of chemokine ligands and receptors [34]. In order to further understand how ASFV manipulates the host chemokines, the chemokine-related factors differentially expressed in RNA-seq were verified. The current data suggested that the transcriptional level of *CCL4* (Fig. 6H), *CCL5* (Fig. 6I), *CXCL8* (Fig. 6J), and *CXCL10* (Fig. 6K) were upregulated to varying degrees after ASFV infection. *CCL4* is a pro-inflammatory chemokine that promotes the development of lymphocytes, which produce IFN-γ [35]. *CCL4* and *CXCL10* have a chemotactic effect on CD4^+^T cells. CXCL8 is the primary mediator of an inflammatory response, attracting neutrophils, basophils, NK cells, and T cells [36]. In addition, some studies have shown that the expression level of CXCL8 and CXCL10 in macrophages infected with low virulent strain OURT88/3 of ASFV was higher than that infected with a virulent strain, which might be crucial for the production of protective immunity in pigs infected with OURT88/3 [36]. The increased level of chemokine transcription in ASFV-infected macrophages might enhance virus clearance by recruiting inflammatory cells. On the other hand, it may also promote the replication of virus in the body by recruiting vulnerable macrophages. Interestingly, the transcriptional level of chemokine receptors *CXCR2* (Fig. 6N) is downregulated after ASFV infection. How a large number of chemokines participate in the process of ASFV infection needs to be explored further.

Apoptosis is vital mechanism for host cells to clear the infection, limit virus replication and reduce virus production in offspring. ASFV, like other viruses, can trigger apoptosis after infection [37]. Presently, many studies have explored the mechanism used by ASFV to trigger apoptosis. Some studies suggested that the fusion of the ASFV virus membrane with intima or virus de-coating is involved in the initial apoptosis induction [38]. Another study reported that the underlying mechanism of inducing apoptosis involves the interaction between ASFV structural protein E183L/p54 and the dynamic protein light chain (DLC8) [39]. In addition, endoplasmic reticulum stress has a major important role in apoptosis induced by ASFV in the later stage of infection, which promotes apoptosis may be beneficial to virus transmission [37]. A179L, a Bcl-2 homologous gene encoded by ASFV, is an effective apoptosis inhibitor that participates in autophagy regulation [40]. The ASFV IAP protein A224L participates in the regulation of apoptosis by inhibiting caspase activation [41]. In addition, ASFV protein EP153R inhibits the induction of apoptosis [42]. Using RNA-seq and qPCR data, we showed that the transcriptional levels of pro-apoptotic and anti-apoptotic factors changed after ASFV infection (Fig. 7). TNFSF10, induces apoptosis of CD4 + and CD8 + T cells [43] and is upregulated, which might explain the cause of lymphopenia in the process of ASFV infection. However, how the apoptosis process develops and what unknown viral proteins participate in ASFV-infected PAMs needs to be explored further.

There are many aspects of the study on the regulation of host transcription by virus. The interaction between ASFV and host nucleus controls controlling host transcription and establishes productive infection [44]. Some studies have confirmed that ASFV similar to other dsDNA viruses has an early stage of intranuclear replication [45]. ASFV infection activates the DNA damage response (DDR) pathway, and ATM-Rad 3 related (ATR) pathway plays an crucial role in ASFV infection [46, 47]. In addition, ASFV infection alters the subnuclear domain and relocate ATR-related factors, to promote heterochromatin, which may regulate transcription and promote virus replication [48]. However, the specific mechanism of ASFV controlling host transcription needs to be elucidated further.

## Conclusions

In summary, we identified the overall transcriptional changes in ASFV-CN/GS/2018 infected PAMs for the first time. Extensive transcriptome and related experimental studies have shown that ASFV-CN/GS/2018 infection leads to leads to changes of PRRs transcription in some RLR and TLR signaling pathways, as well as the significant changes of transcriptional of some anti-viral and inflammatory factors. In addition, ASFV-CN/GS/2018 infection is involved in the regulation of chemokine expression in PAMs, such as CXCL8 and CXCL10. At the same time, we found that ASFV-CN/GS/2018 may be involved in the regulation of host apoptosis in many ways; the transcriptional levels of pro-apoptotic and anti-apoptotic factors changed after infection. These studies provide a necessary reference for deepening the understanding of host response after ASFV-CN/GS/2018 infection and effective information for screening candidate targets for ASFV inhibition. In this study, the transcriptional changes of PAMs infected by ASFV-CN/GS/2018 were explained as a whole, and the possible biological processes involved in ASFV-CN/GS/2018 infection were preliminarily explored. Yet, the specific mechanism of ASFV involved in host cell-related biological processes needs to be further investigated.

## Supplementary Information

**Additional file 1**. Heat map of differentially expressed genes.

**Additional file 2.** ASFV 12h vs. MOCK 12h. Information of differentially expressed genes at 12 hpi.

**Additional file 3**. ASFV 24h vs. MOCK 24h. Information of differentially expressed genes at 24 hpi.

**Additional file 4**. ASFV 36h vs. MOCK 36h. Information of differentially expressed genes at 36 hpi.

## Data Availability

All data generated or analyzed during this study are included in this submitted.

## Abbreviations

ASFV: African swine fever virus
PAMs: Porcine alveolar macrophages
PCA: Principal component analysis
BMP8A: Bone morphogenetic protein 8B
HES4: Transcription factor HES-4
GPR37: Prosaposin receptor GPR37
AQP9: Aquaporin-9
IFIT1: Interferon-induced protein with tetratricopeptide repeats 1
RSAD2: Radical S-adenosyl methionine domain-containing protein 2
CXCL8: C-X-C motif chemokine 8
PLAC8: Placenta-specific gene 8 protein
ARG1: Arginase-1
PRR: Pattern recognition receptors
KEGG: Kyoto Encyclopedia of Genes and Genomes
TLR3: Toll-like receptor 3
DDX58: DEAD box polypeptide 58
IFIH1: Interferon-induced helicase C domain-containing protein 1
IFIT1: Interferon-induced protein with tetratricopeptide repeats 1
IFITM3: Interferon-induced transmembrane protein 3
IL-6: Interleukin-6
TNF-α: Tumor necrosis factor α
NF-κB: Nuclear factor NF-kappa-B p50 subunit
*CCL4*: C-C motif chemokine 4
TNFSF10: Tumor necrosis factor ligand superfamily member 10
IFI6: Interferon alpha-inducible protein 6
MOI: Multiplicity of infection
HAD_50_: 50% Hemadsorption doses
qPCR: Quantitative real-time polymerase chain reaction
DMEM: Dulbecco’s modified Eagle’s medium
FBS: Fetal bovine serum
ETAA1: Ewing's tumor-associated antigen 1 homolog
GPR37: Prosaposin receptor GPR37
CXCR2: C-X-C chemokine receptor type 2
PIK3CB: Phosphatidylinositol 4, bisphosphate 3-kinase catalytic subunit beta isoform
HRK: Activator of apoptosis harakiri
