# Correction to: Mechanism of interaction between virus and host is inferred from the changes of gene expression in macrophages infected with African swine fever virus CN/GS/2018 strain

**DOI:** 10.1186/s12985-021-01654-5

**Published:** 2021-09-13

**Authors:** Bo Yang, Chaochao Shen, Dajun Zhang, Ting Zhang, Xijuan Shi, Jinke Yang, Yu Hao, Dengshuai Zhao, Huimei Cui, Xingguo Yuan, Xuehui Chen, Keshan Zhang, Haixue Zheng, Xiangtao Liu

**Affiliations:** State Key Laboratory of Veterinary Etiological Biology, National Foot‑and‑Mouth Disease Reference Laboratory, Lanzhou Veterinary Research Institute, Chinese Academy of Agriculture Science, Lanzhou, 73004 China

## Correction to: Virol J (2021) 18:170 https://doi.org/10.1186/s12985-021-01637-6

Following publication of the original article [1], the authors informed us that there is an inconsistency in Figure 1C, between the online version and the PDF version.

The PDF version has been corrected.

The original article has been corrected.
