# Supplementary figures and images for: Mechanism of interaction between virus and host is inferred from the changes of gene expression in macrophages infected with African swine fever virus CN/GS/2018 strain
Source: Virol J. 2021 Aug 19;18:170. doi: 10.1186/s12985-021-01637-6 (PMC8375147; doi:10.1186/s12985-021-01637-6)

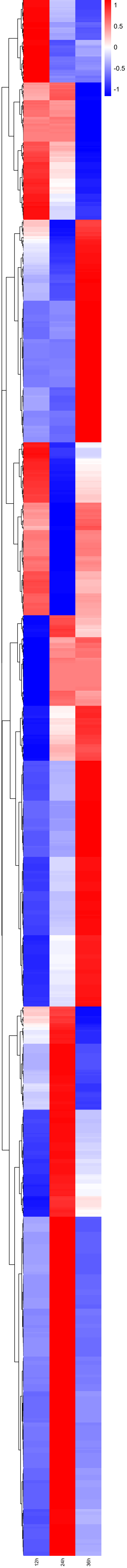

Supplement: Supplementary file 1 — Additional file 1. Heat map of differentially expressed genes. [file 12985_2021_1637_MOESM1_ESM.pdf]
